# Mosaic loss of chromosome Y promotes leukemogenesis and clonal hematopoiesis

**DOI:** 10.1172/jci.insight.153768

**Published:** 2022-02-08

**Authors:** Qi Zhang, Lei Zhao, Yi Yang, Shujun Li, Yu Liu, Chong Chen

**Affiliations:** Department of Hematology and Institute of Hematology, State Key Laboratory of Biotherapy and Cancer Center, West China Hospital, Sichuan University, Chengdu, China.

**Keywords:** Genetics, Hematology, Genetic instability, Leukemias

## Abstract

Mosaic loss of chromosome Y (mLOY) in blood cells is one of the most frequent chromosome alterations in adult males. It is strongly associated with clonal hematopoiesis, hematopoietic malignancies, and other hematopoietic and nonhematopoietic diseases. However, whether there is a causal relationship between mLOY and human diseases is unknown. Here, we generated mLOY in murine hematopoietic stem and progenitor cells (HSPCs) with CRISPR/Cas9 genome editing. We found that mLOY led to dramatically increased DNA damage in HSPCs. Interestingly, HSPCs with mLOY displayed significantly enhanced reconstitution capacity and gave rise to clonal hematopoiesis in vivo. mLOY, which is associated with *AML1-ETO* translocation and p53 defects in patients with acute myeloid leukemia (AML), promoted AML in mice. Mechanistically, loss of KDM5D, a chromosome Y–specific histone 3 lysine 4 demethylase in both humans and mice, partially recapitulated mLOY in DNA damage and leukemogenesis. Thus, our study validates mLOY as a functional driver for clonal hematopoiesis and leukemogenesis.

## Graphical abstract

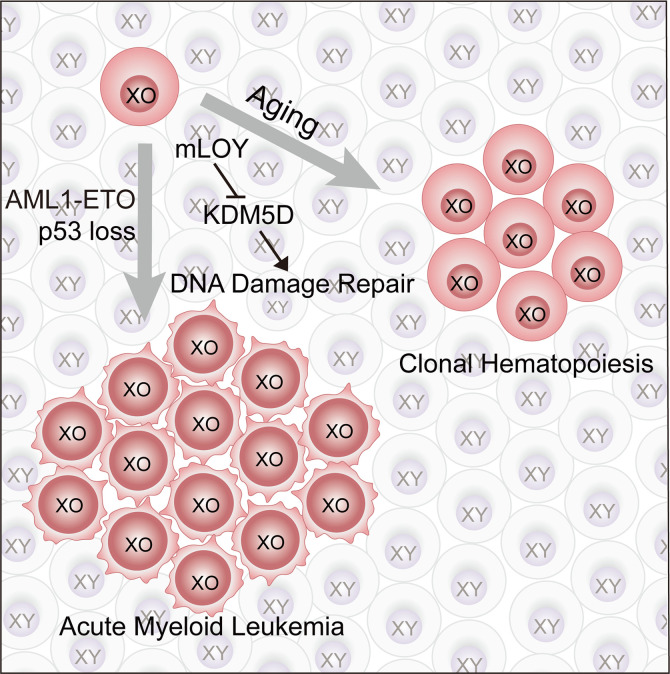

## Introduction

Mosaic loss of chromosome Y (mLOY) is the most frequent chromosome alteration in adult males’ blood cells (1, 2). It is estimated that approximately 20% of the male population has detectable mLOY in peripheral blood cells (3). With aging, the frequency of mLOY in the population and the levels of mLOY are dramatically increased. More than 70% of the elderly population has blood cells with mLOY, and up to 100% of their blood cells may lose chromosome Y (3). Moreover, it has been shown that mLOY is tightly associated with multiple hematopoietic and nonhematopoietic human diseases, such as clonal hematopoiesis, leukemia, solid cancers, Alzheimer’s disease, cardiovascular events, and eventually all-cause mortality (4–10). Clonal hematopoiesis (CH) is a common aging-related hematopoietic abnormality in which a single hematopoietic stem or progenitor cell gives rise to a substantial proportion of peripheral blood cells or bone marrow cells (hematopoietic stem and progenitor cells, HSPCs) (11). mLOY has been suggested as a marker for the diagnosis of clonal hematopoiesis (6, 12–15). Furthermore, mLOY in leukocytes is a predisposition for hematopoietic malignancies, including acute myeloid leukemia (AML), acute lymphoid leukemia, and myelodysplastic syndrome (4, 16). Among them, up to 60% of AML with t(8;21) (q22;q22) (*AML1-ETO*) showed mLOY (17–20). However, due to the technical challenges to precisely deleting a whole chromosome in somatic cells, functional evidence of mLOY in hematopoietic cells is still missing.

XO mice had been applied to study the role of chromosome Y in leukemogenesis but failed to recapitulate the pathology of mLOY in patients (21). One potential explanation might be the different DNA damage responses in embryonic stem cells and somatic cells (22, 23). Consistently, accumulating evidence indicates that Turner syndrome patients have a reduced risk of cancer, including hematopoietic malignancies, which is in sharp contrast to the strong association of mLOY with multiple cancers in aging males (24, 25). Thus, we asked whether mLOY in somatic cells, but not XO in the germline, would drive malignant transformation.

Fortunately, recent advances in genome editing, including CRISPR/Cas9, have made it possible to remove an entire chromosome precisely (26–29). In this study, we generated mLOY in HSPCs with CRISPR/Cas9 and investigated its potential functions in AML and CH.

## Results

### Generating mLOY in murine HSPCs.

**Figure 1:**
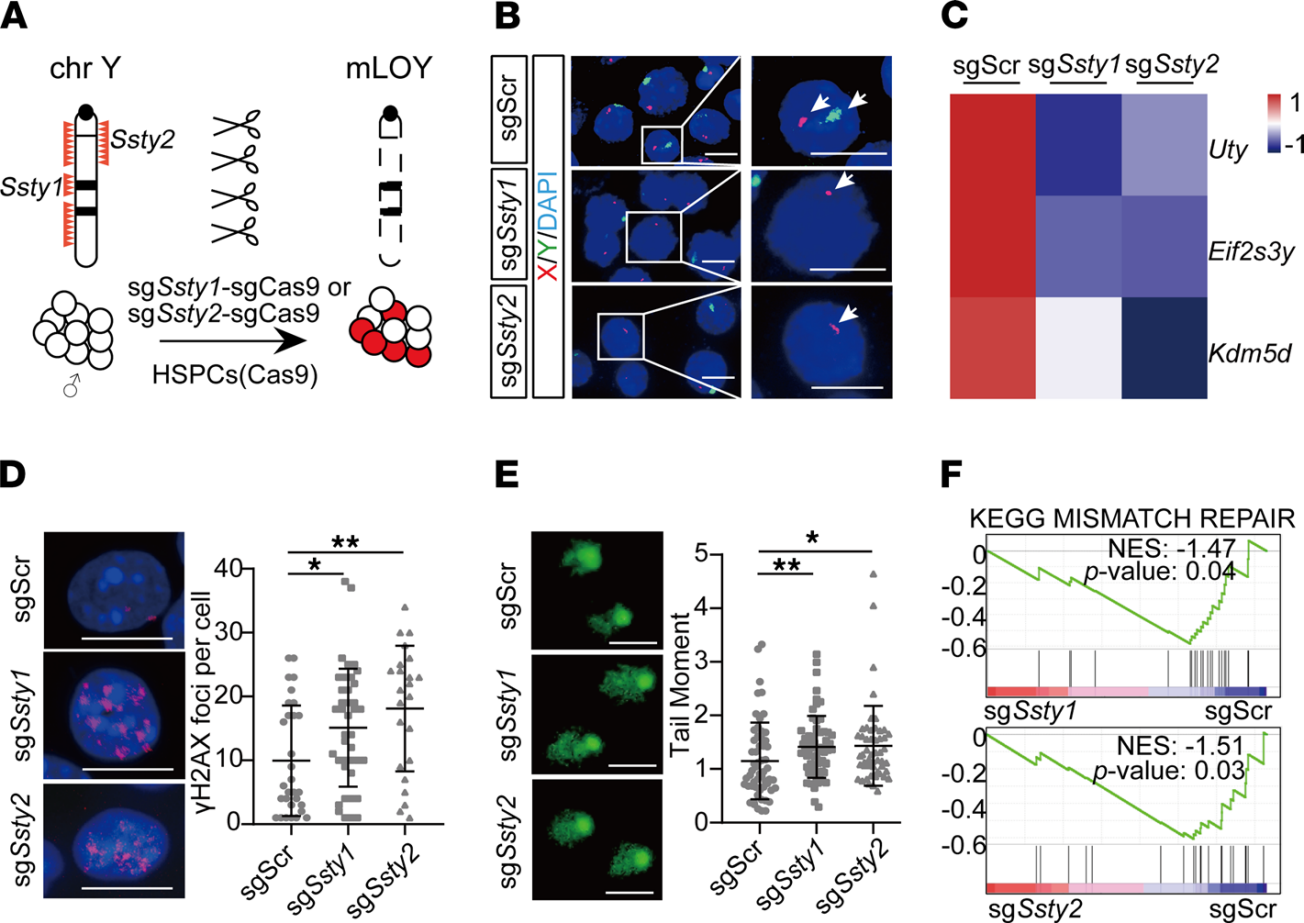
Generating mLOY in mouse HSPCs. (**A**) Schematic of CRISPR/Cas9 genome editing of chromosome Y in mouse HSPCs. sg*Ssty1*-sgCas9 and sg*Ssty2*-sgCas9 target repeat sequences located on chromosome Y, which causes chromosome Y elimination. (**B**) FISH analysis of chromosomes X and Y in sgScr-sgCas9, sg*Ssty1*-sgCas9, and sg*Ssty2*-sgCas9 HSPCs. Green, FITC-labeled whole-chromosome probe for Y chromosome; red, Texas red–labeled X chromosome probe for XqA7.3; blue, DAPI-labeled DNA. White arrows indicate chromosomes X and Y. Squares indicate single cells shown at a higher resolution in the right panels. Scale bars: 10 μm. (**C**) Heatmap showing the relative expression levels of the chromosome Y–specific genes in sg*Ssty1*-sgCas9 and sg*Ssty2*-sgCas9 HSPCs compared with sgScr-sgCas9 HSPCs, measured by RNA-seq 8 days after infection (*n* = 3 for each group). (**D**) Left: Representative immunofluorescence images of γH2AX foci in sgScr-sgCas9, sg*Ssty1*-sgCas9, and sg*Ssty2*-sgCas9 HSPCs. HSPCs were cultured 1 month in vitro until Cas9 elimination. Scale bars: 10 μm. Red, γH2AX; blue, DAPI-labeled DNA. Right: Plot of γH2AX foci per cell, shown as the mean ± SD. *FDR *q* < 0.05, **FDR *q* <0.01 (Kruskal-Wallis test). (**E**) Left: Representative images of comet assay of sgScr-sgCas9, sg*Ssty1*-sgCas9, and sg*Ssty2*-sgCas9 HSPCs. HSPCs were cultured 1 month in vitro until Cas9 elimination. Scale bars: 50 μm. Right: Results of comet assay of sgScr-sgCas9, sg*Ssty1*-sgCas9, and sg*Ssty2*-sgCas9 HSPCs. The tail moment is shown as the mean ± SD. *FDR *q* < 0.05, **FDR *q* < 0.01 (Kruskal-Wallis test). (**F**) GSEA showing the negative enrichment of the KEGG_MISMATCH_REPAIR gene set in sg*Ssty1*-sgCas9 HSPCs (NES = –1.47; *P* = 0.04) (top) and sg*Ssty2*-sgCas9 HSPCs (NES = –1.51; *P* = 0.03) (bottom).

Taking advantage of recent advances in genome editing (27), we designed a strategy to generate mLOY by introducing 2 independent single guide RNAs (sgRNAs) targeting *Ssty1* (spermiogenesis-specific transcript on Y 1) or *Ssty2* (spermiogenesis-specific transcript on Y 2) in c-Kit^+^ HSPCs from Rosa-Cas9 mice (Figure 1A). Because there are more than 300 copies of *Ssty1* and *Ssty2* repeats specifically located on chromosome Y, multiple cuts by sgRNAs on the same chromosome would result in the deletion of the whole chromosome, at least in embryos (27, 29). Previous whole-genome sequencing showed that these sgRNAs had minimal off-target effects and could give rise to healthy animals without chromosome Y (27). To further exclude the possibility of continuous cutting by CRISPR/Cas9 and thus potential off-target effects, we cointroduced a suicide sgRNA against Cas9, which was linked in tandem with sg*Ssty1* or sg*Ssty2* (Supplemental Figure 1A; supplemental material available online with this article; https://doi.org/10.1172/jci.insight.153768DS1). By immunofluorescence staining, we found that sgCas9 completely depleted Cas9 in HSPCs (Supplemental Figure 1B).

These sg*Ssty1*-sgCas9 and sg*Ssty2*-sgCas9 HSPCs were analyzed by fluorescence in situ hybridization (FISH) with FITC-labeled whole–chromosome Y probes to visualize chromosome Y loss with Texas Red–labeled chromosome XqA7.3 probes as staining control, with sgScr-sgCas9 cells as control (27). Although all of the control HSPCs had intact Y chromosomes, approximately 15% of sg*Ssty1*-sgCas9 and sg*Ssty2*-sgCas9 HSPCs displayed mLOY, as indicated by negative staining of the chromosome Y probes (Figure 1B). Transcriptome analyses showed that the expression levels of chromosome Y–specific genes, *Uty, Eif2s3y*, and *Kdm5d*, were significantly reduced in sg*Ssty1*-sgCas9 and sg*Ssty2*-sgCas9 HSPCs compared with those with sgScr-sgCas9 (Figure 1C). Thus, sg*Ssty1* and sg*Ssty2* successfully created mLOY in HSPCs.

### mLOY leads to increased DNA damage in HSPCs.

It has been reported that mLOY is tightly associated with chromosome instability in human blood cells (3, 8, 12). Although it has been proposed that mLOY might be a consequence of genomic instability, it is also possible that mLOY might be a cause of genomic instability. To test the latter hypothesis, we infected Cas9 HSPCs with sg*Scr*-sgCas9, sg*Ssty1*-sgCas9, or sg*Ssty2*-sgCas9 and then analyzed the ratio of mLOY cells to non-mLOY cells by FISH and DNA damage by the comet assay and γH2AX staining over time. First, we observed an increase in the mLOY ratio from 3 days to 15 days after infection, which suggested that HSPCs with mLOY might have a growth advantage over control cells (Supplemental Figure 1C). Immunofluorescence staining showed that both sg*Ssty1*-sgCas9 and sg*Ssty2*-sgCas9 HSPCs had significantly higher levels of γH2AX than the sgScr-sgCas9 HSPCs (Figure 1D). Consistently, there was significantly increased DNA damage, as indicated by the tail moment, in sg*Ssty1*-sgCas9 and sg*Ssty2*-sgCas9 HSPCs compared with the control cells (Figure 1E and Supplemental Figure 1D). To further rule out the potential off-target effects of sg*Ssty1* and sg*Ssty2*, we introduced them into HSPCs from female Cas9 mice. The results showed that there was no significant difference in sg*Ssty1*-sgCas9 and sg*Ssty2*-sgCas9 compared with sgScr-sgCas9 in these XX cells (Supplemental Figure 1D). Taken together, our results show that mLOY itself led to DNA damage in HSPCs.

Consistently, gene set enrichment analysis (GSEA) showed that the KEGG_MISMATCH_REPAIR pathway was significantly negatively enriched in sg*Ssty1*-sgCas9 (normalized enrichment score [NES] = –1.47, *P* = 0.04) and sg*Ssty2*-sgCas9 HSPCs (NES = –1.51, *P* = 0.03) compared with sgScr-sgCas9 cells (Figure 1F). These data strongly suggest that mLOY gave rise to increased levels of DNA damage in HSPCs.

### mLOY accelerates leukemogenesis.

mLOY is strongly associated with a high risk of leukemia (6, 12, 30). Previous meta-analyses showed that approximately half of *AML1-ETO+* AML had mLOY (12, 17, 20). To further investigate the mLOY spectrum in AML, we analyzed 3 independent AML cohorts with a total of 620 patients. In the TARGET AML cohort, 16 out of the total 250 male patients showed mLOY and 14 of them (87.5%) were also *AML1-ETO*^+^. And conversely, 35.9% (14/39) of *AML1-ETO*^+^ AML were also mLOY (31). Thus, mLOY was significantly correlated with *AML1-ETO* in the TARGET AML cohort (*P* = 2.6 × 10^–16^) (Supplemental Figure 2A). Similarly, we also observed a strong association in the BEAT AML and The Cancer Genome Atlas (TCGA) LAML cohorts (32, 33) (*P* = 1.5 × 10^–11^ and *P* = 1.1 × 10^–15^, respectively) (Supplemental Figure 2, B and C). In TCGA LAML cohort, 2 of 5 AML with mLOY also had mutations in, or deletion of, *TP53* (Supplemental Figure 3A). In the BEAT AML cohort, 5 of 15 AML with mLOY also had missense mutations in *TP53* (Supplemental Figure 3B). Further, all of the p53-intact AML patients with mLOY had significant downregulation of the HALLMARK_p53 pathway compared with those without mLOY (TCGA LAML, NES = –1.82, *P* = 0.00; BEAT AML, NES = –1.33, *P* = 0.01) (Supplemental Figure 3, C and D). These data suggest that mLOY was associated with both *AML1-ETO* translocation and *TP53* deficiency in AML.

**Figure 2:**
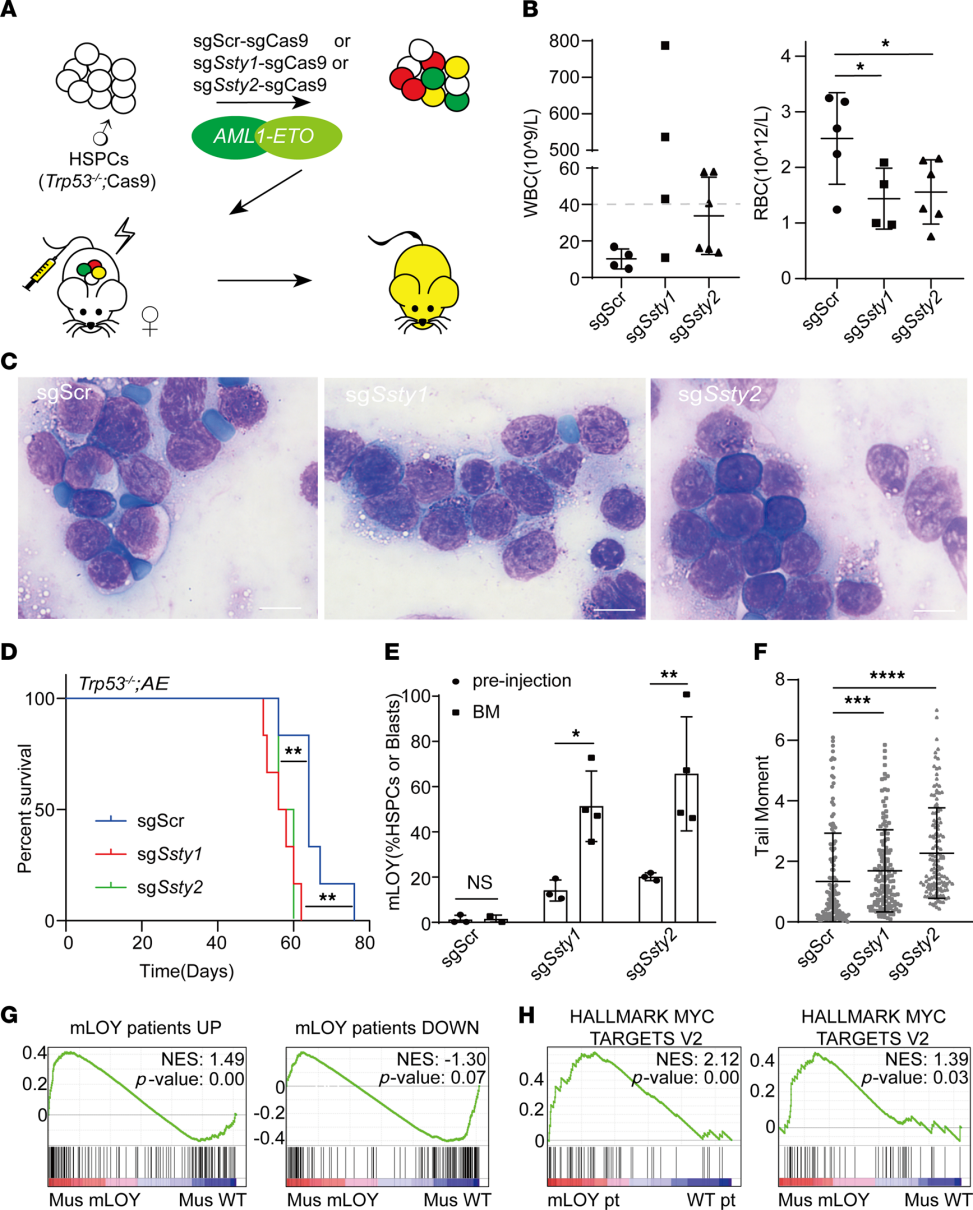
mLOY collaborates with *AML1-ETO* to promote leukemogenesis. (**A**) Schematic of mLOY AML mouse model. HSPCs from male *Trp53–/–*; Cas9 mice were infected with mCherry-linked Y-chromosome-targeting sgRNA (sg*Ssty1* and sg*Ssty2*)-sgCas9 and GFP-linked *AML1-ETO* and then transplanted into sublethally irradiated female recipient mice, with sgScr-sgCas9 as a negative control. (**B**) White blood cell (WBC) counts and red blood cell (RBC) counts of recipient mice at 7 weeks after transplantation with sgScr-sgCas9; *AML1-ETO*, sg*Ssty1*-sgCas9; *AML1-ETO*, and sg*Ssty2*-sgCas9; *AML1-ETO* HSPCs. WBC counts greater than 40 × 10^9^/L are above the gray dotted line and those less than 40 × 10^9^/L are below. Data shown as mean ± SD. *n* = 4, 4, 6 (WBC); *n* = 5, 4, 6 (RBC). *FDR *q* < 0.05 (1-way ANOVA). (**C**) Representative images of blood smear at 7 weeks from recipient mice. Scale bars: 10 μm. (**D**) Kaplan-Meier tumor-free survival curves of recipient mice (*n* = 6). **P < 0.01 (log-rank test). (**E**) The frequencies of mLOY in HSPCs before injection and blast cells in AML mouse tumor cells; tumor cells were harvested from bone marrow at each of the endpoints when recipient mice developed full-blown AML (BM). Data are shown as mean ± SD. *n* = 3, 2 (preinjection), *n* = 3, 4 (sg*Ssty1*), *n* = 3, 4 (sg*Ssty2*). NS, not significant, *P < 0.05, **P < 0.01 (2-way ANOVA). (**F**) Comet assay of AML tumor cells (3 mice per group). The tail moment is shown as the mean ± SD. ***FDR *q* < 0.001, ****FDR *q* < 0.0001 (Kruskal-Wallis test). (**G**) GSEA showing the enrichment of gene signatures (top 200 differentially expressed genes upregulated or downregulated) of mLOY AML patients and mLOY AML mice compared with WT (mLOY and WT patients were derived from the *AML1-ETO* samples in the TARGET-AML cohort). (**H**) GSEA showing the enrichment of the HALLMARK_MYC_TARGET_V2 pathway in both mLOY AML patients and mLOY AML mice compared with WT (mLOY pt, AML patients with mLOY; WT pt, AML patients with intact Y chromosome).

To investigate the function of mLOY in leukemogenesis, we cotransduced sg*Ssty1*-sgCas9, sg*Ssty2*-sgCas9, or sgScr-sgCas9 and *AML1-ETO* into *Trp53–/–*; Cas9 HSPCs and transplanted them into sublethally irradiated wild-type (WT) recipient mice (Figure 2A). All recipients were monitored by complete blood count (CBC) assay and blood smear for leukemogenesis. At 7 weeks after transplantation, 3 out of 4 sg*Ssty1*-sgCas9; *AML1-ETO* recipients and 3 out of 6 sg*Ssty2*-sgCas9; *AML1-ETO* recipients had white blood cell (WBC) counts greater than 40 × 10^9^/L, while all of the control sgScr-sgCas9; AML1-ETO recipients had WBC counts of approximately 10 × 10^9^/L. sg*Ssty1*-sgCas9; *AML1-ETO* and sg*Ssty2*-sgCas9; *AML1-ETO* mice also had significantly reduced red blood cell (RBC) counts compared with the control mice (Figure 2B). Leukemic blasts were observed in the peripheral blood of all recipient mice (Figure 2C). The platelet numbers in their peripheral blood were similar (Supplemental Figure 4A). While the sgScr-sgCas9; *AML1-ETO* recipients developed AML with an average latency of 64 days, both sg*Ssty1*-sgCas9; *AML1-ETO* and sg*Ssty2*-sgCas9; *AML1-ETO* mice developed AML with significantly shorter latency (56 days and 56 days, respectively) (Figure 2D). Although mLOY mice were harvested earlier than control mice, their liver and spleen weights were similar to those of the control animals (Supplemental Figure 4, B and C). Most of the leukemic cells from either group expressed high levels of c-Kit and myeloid markers Gr-1 and/or Mac-1, but not lymphoid marker CD3 or B220 (Supplemental Figure 4E). These results indicate that all of these mice developed full-blown AML.

Then, we analyzed the genetics and cytogenetics of the resulting AML cells. Consistently with the preleukemic HSPCs in vitro, sgCas9 completely depleted the expression of Cas9 in the resulting sg*Ssty1*-sgCas9; *AML1-ETO* and sg*Ssty2*-sgCas9; *AML1-ETO* AML cells (Supplemental Figure 4D). FISH analyses showed that 50% of sg*Ssty1*-sgCas9; *AML1-ETO* and 65% of sg*Ssty2*-sgCas9; *AML1-ETO* leukemic cells had lost their Y chromosome, more than a 4-fold increase from their initial mLOY ratio before transplantation (Figure 2E and Supplemental Figure 4F). Cytogenetic analyses confirmed the complete loss of chromosome Y and a largely normal karyotype of the other chromosomes in sg*Ssty2* mLOY leukemic cells (Supplemental Figure 4G). Consistent with mLOY HSPCs, mLOY AML cells directly harvested from sick mice displayed significantly higher levels of ongoing DNA damage in leukemic cells, as measured by the comet assay (Figure 2F).

Further, we wondered whether mouse mLOY AML would recapitulate the molecular signature of human AML with mLOY. RNA sequencing (RNA-seq) analyses showed that the upregulated and downregulated gene sets in human AML with mLOY were significantly positively and negatively, respectively, enriched in mouse mLOY AML (UP: NES = 1.49, *P* = 0.00; DOWN: NES = –1.30, *P* = 0.07) (Figure 2G). Human and mouse mLOY AML also shared common pathways important for leukemogenesis, such as the HALLMARK_MYC_TARGETS_V2 pathway (human: NES = 2.12, *P* = 0.00; mouse: NES = 1.39, *P* = 0.03) (Figure 2H). The similarity of the transcriptomes of mouse and human AML with mLOY suggested a common molecular mechanism underlying mLOY-driven AML in humans and mice, although the synteny between mouse and human Y chromosomes is poor (34).

### mLOY promotes CH in mice.

**Figure 3:**
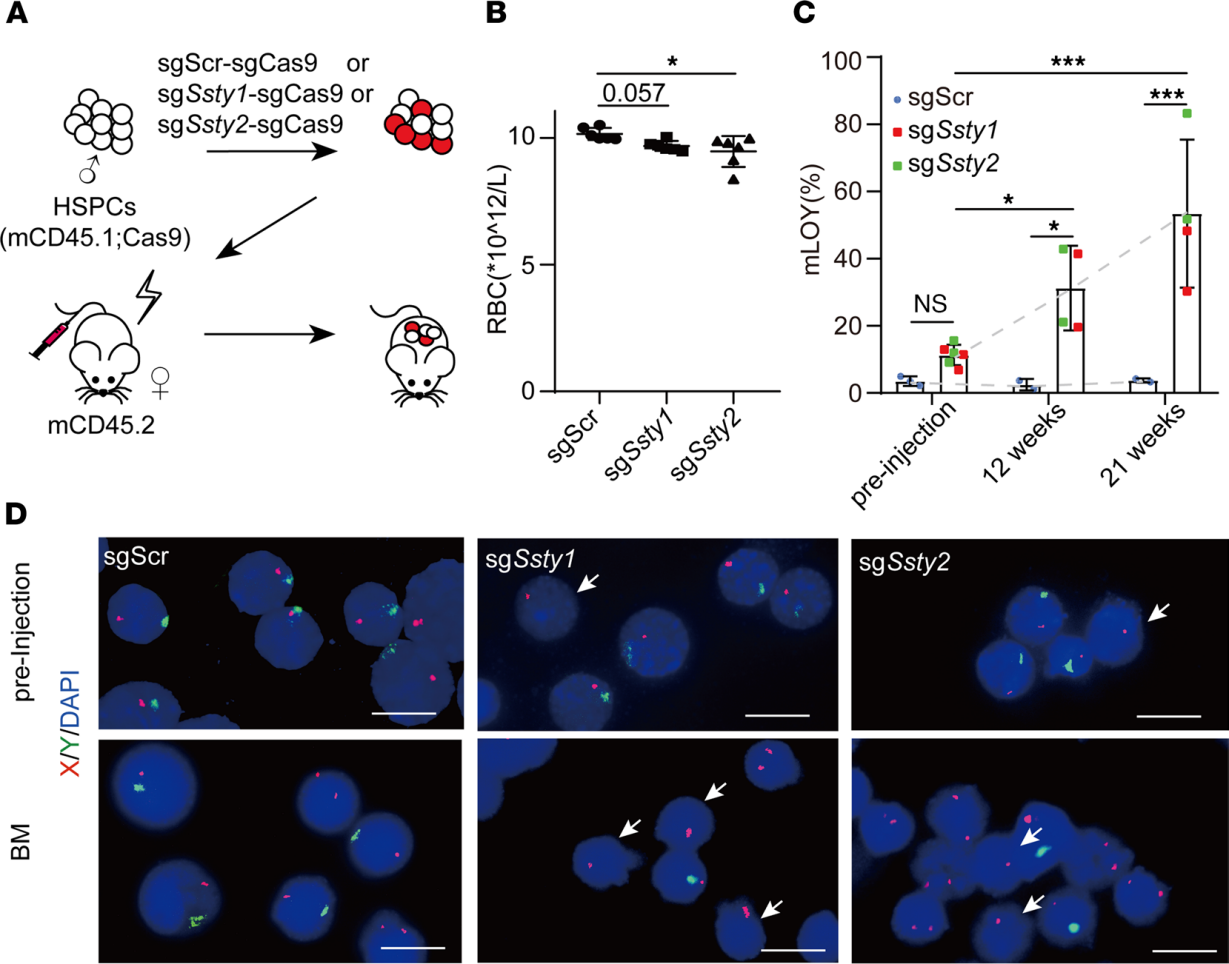
mLOY gives rise to clonal hematopoiesis in mice. (**A**) Schematic showing the experimental design. HSPCs from male mCD45.1; Cas9 mice were infected with mCherry-linked Y-chromosome-targeting sgRNA (sg*Ssty1* and sg*Ssty2*)-sgCas9 and then transplanted into sublethally irradiated (7 Gy) mCD45.2 female recipient mice, with sgScr-sgCas9 as a negative control. (**B**) RBC counts of recipient mice 9 weeks after transplantation with sgScr-sgCas9, sg*Ssty1*-sgCas9, and sg*Ssty2*-sgCas9 HSPCs. Data shown as mean ± SD (*n* = 6). *FDR *q* < 0.05 (1-way ANOVA). (**C**) Percentages of mLOY in sgScr-sgCas9, sg*Ssty1*-sgCas9, and sg*Ssty2*-sgCas9 HSPCs before injection and donor-derived bone marrow cells at 12 weeks and 21 weeks after transplantation in recipient mice. The gray dotted line shows the growth trend. *n* = 3, 6 (preinjection), *n* = 2, 4 (12 weeks), *n* = 2, 4 (21 weeks). NS, not significant, *P < 0.05, ***P < 0.001 (2-way ANOVA). (**D**) Representative photomicrographs of FISH of sgScr-sgCas9, sg*Ssty1*-sgCas9, and sg*Ssty2*-sgCas9 HSPCs before injection (top) and bone marrow cells from recipient mice that developed full-blown AML (BM, bottom). Green, FITC-labeled whole-chromosome probe for Y chromosome; red, Texas red–labeled X chromosome probe for XqA7.3; blue, DAPI-labeled DNA. White arrows indicate XO cells. Scale bars: 10 μm.

mLOY is associated with CH in elderly males (3, 30). We tested the potential function of mLOY in CH by transfusing HSPCs from young adult Rosa-Cas9 mice infected with sg*Ssty1*-sgCas9, sg*Ssty2*-sgCas9, or control sgScr-sgCas9 HSPCs, together with uninfected cells into sublethally irradiated (7 Gy) congenic WT recipient mice (Figure 3A). There were no significant differences in the numbers of WBCs and the ratios of B and myeloid cells between the recipients with sg*Ssty1*-sgCas9, sg*Ssty2*-sgCas9, or control sgScr-sgCas9 HSPCs (Supplemental Figure 5, A and B). However, the CBC assay showed that there were mild but significant reductions in RBC counts in the peripheral blood of both sg*Ssty1*-sgCas9 and sg*Ssty2*-sgCas9 mice compared with those of the control mice, which was consistent with frequent anemia in CH patients (35) (Figure 3B).

Importantly, FISH analyses showed that there were significantly increased ratios of cells with mLOY in the bone marrow of both sg*Ssty1*-sgCas9 and sg*Ssty2*-sgCas9 mice over time. There were minimal mLOY cells in sgScr-sgCas9 mice. In contrast, there were approximately 30% mLOY cells in the sg*Ssty1*-sgCas9 and sg*Ssty2*-sgCas9 mice at 12 weeks after transplantation, and more than 50% mLOY cells at 21 weeks, respectively, compared with approximately 15% mLOY in preinjected cells (Figure 3, C and D). The expansion of these mLOY cells in the recipients and associated anemia resembled CH in elderly males.

### KDM5D loss mediates the function of mLOY in AML.

**Figure 4:**
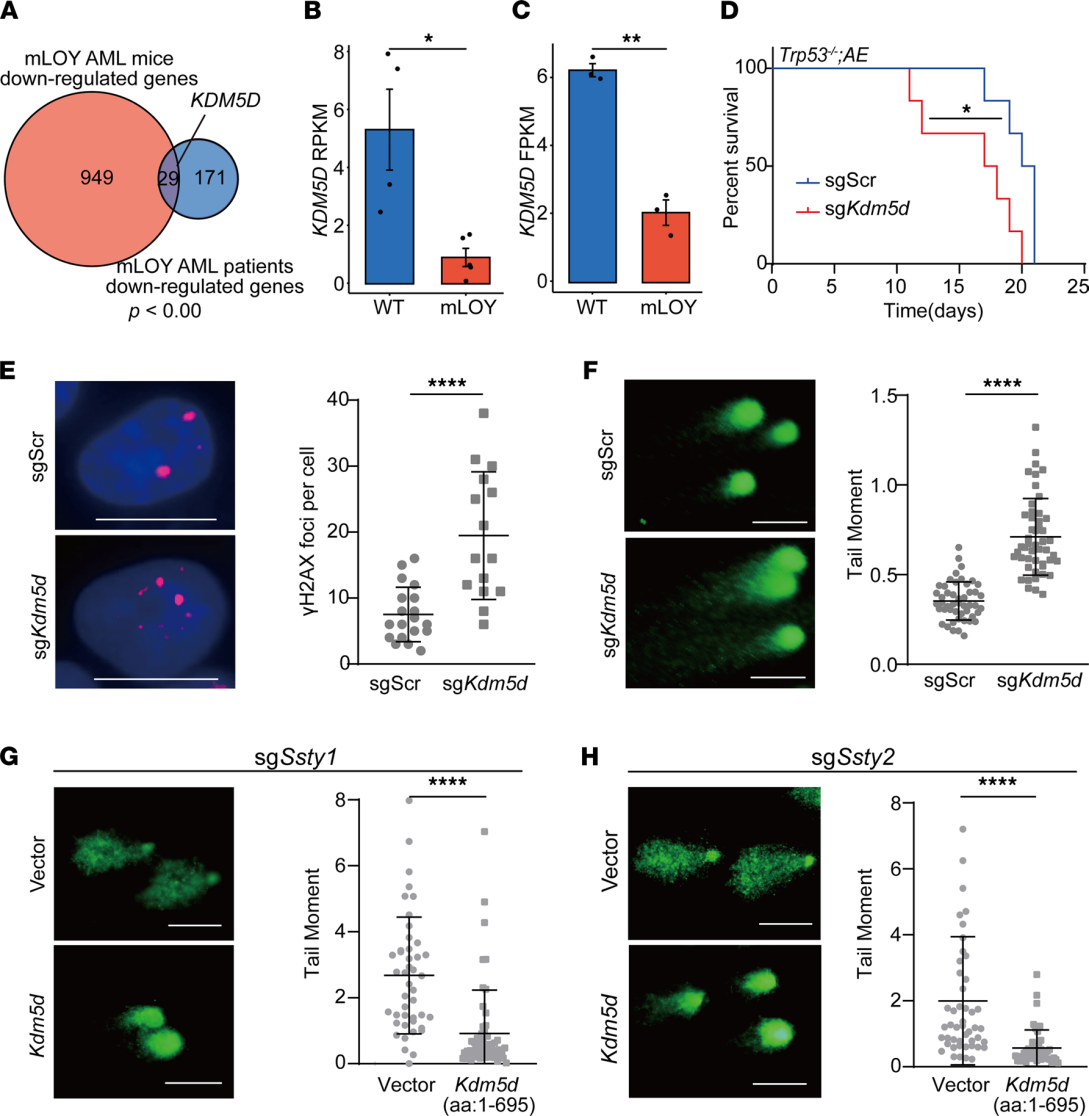
*KDM5D* loss promotes tumorigenesis and increased DNA damage. (**A**) Venn diagram showing the enrichment of the significantly downregulated genes in mLOY AML patients (top 200) and AML mice. (**B** and **C**) The expression levels of *KDM5D* in mLOY AML patients with *AML1-ETO* (**B**, *n* = 5; **C**, *n* = 3) and other *AML1-ETO+* patients (**B**, *n* = 4; **C**, *n* = 3) in 2 AML cohorts (TARGET AML and BEAT AML), from analysis of The Cancer Genome Atlas data. *P < 0.05, **P < 0.01 (2-tailed *t* test). (**D**) Kaplan-Meier tumor-free survival curve of recipient mice with sgScr; *AML1-ETO* and sg*Kdm5d*; *AML1-ETO* AML cells (*n* = 6). *P < 0.05 (log-rank test). (**E**) Left: Representative immunofluorescence images of γH2AX foci in sgScr and sg*Kdm5d* HSPCs. Scale bars: 10 μm. Red, γH2AX; blue, DAPI-labeled DNA. Right: Plot of γH2AX foci per cell, shown as the mean ± SD. ****P < 0.0001 (2-tailed Mann-Whitney test). (**F**) Left: Representative images of comet assay of sgScr and sg*Kdm5d* HSPCs. Scale bars: 50 μm. Right: Plot of comet assay of sgScr and sg*Kdm5d* HSPCs. The tail moment is shown as the mean ± SD. ****P < 0.0001 (2-tailed Mann-Whitney test). (**G**) Left: Representative images of comet assay of sg*Ssty1-*sgCas9 HSPCs with truncated-*Kdm5d* overexpression (bottom, the truncated KDM5D is a 659–amino acid protein consisting of aa 1–695) and vector only (top) was used as a negative control. Scale bars: 50 μm. Right: Plot of comet assay of sg*Ssty1*-sgCas9 HSPCs with truncated-*Kdm5d* overexpression and vector only. The tail moment is shown as the mean ± SD. ****P < 0.0001 (2-tailed Mann-Whitney test). (**H**) Left: Representative images of comet assay of sg*Ssty2*-sgCas9 HSPCs with truncated-*Kdm5d* overexpression (bottom) and vector only (top). Scale bars: 50 μm. Right: Plot of comet assay of sg*Ssty2-*sgCas9 HSPCs with truncated-*Kdm5d* overexpression and vector only. The tail moment is shown as the mean ± SD. ****P < 0.0001 (2-tailed Mann-Whitney test).

In order to further explore the mechanism of mLOY in AML, we analyzed the commonly downregulated genes in both human and mouse AML with mLOY. Among the 29 common downregulated genes was that encoding lysine demethylase 5D (*KDM5D*), a chromosome Y–specific H3K4 demethylase (Figure 4, A–C).

*KDM5D* had been suggested to be a tumor suppressor in prostate cancer, but its role in hematopoietic malignancies was unknown (36). In AML, low *KDM5D* expression was associated with *AML1-ETO* translocation, similar to mLOY (Supplemental Table 1). To test the function of *KDM5D* in AML, we transplanted *Trp53*^–/–^; *AML1-ETO*; Cas9 leukemic cells with sgScr or sg*Kdm5d* into sublethally irradiated recipient mice. The average survival of sg*Kdm5d* AML mice was 17 days, significantly shorter than that of sgScr AML mice (20 days) (Figure 4D).

Since mLOY led to increased DNA damage in HSPCs, we wondered whether *Kdm5d* loss might mimic mLOY to promote DNA damage. *Kdm5d* was disrupted in HSPCs with CRISPR/Cas9, and γH2AX staining and the comet assay were performed to measure their DNA damage. The results showed that there were significantly more γH2AX foci in the sg*Kdm5d* cells than the control sgScr HSPCs (Figure 4E). Moreover, there were also increased tail moment levels for the sg*Kdm5d* HSPCs compared with those with sgScr (Figure 4F). Further, we ectopically expressed a truncated *Kdm5d* (encoding aa 1–695, including the JmjN, ARID, PHD-type 1, and JmjC domains) in the mLOY HSPCs. The comet assay showed that *Kdm5d* significantly reduced the tail moment of both sg*Ssty1* and sg*Ssty2* HSPCs (Figure 4, G and H). These results strongly suggest that *Kdm5d* plays critical roles in preventing DNA damage and leukemogenesis.

Lastly, we explored the molecular consequences of *Kdm5d* loss in HSPCs by RNA-seq analyses (Supplemental Figure 6A). Consistently with the increased level of DNA damage, the DNA damage checkpoint gene signature was significantly positively enriched in *Kdm5d*-deficient cells. And similarly, human AML with low expression levels of *KDM5D* also had upregulated expression of the DNA damage checkpoint genes (human: NES = 2.69, *P* = 0.00, mouse: NES = 1.85, *P* = 0.00) (Supplemental Figure 6B). *Kdm5d-*deficient HSPCs and low-*KDM5D*-expressing AML patients also shared the common leukemia-promoting HALLMARK_MYC_TARGETS_V2 pathway (human: NES = 1.88, *P* = 0.00; mouse: NES = 2.07, *P* = 0.00), which was consistent with those in both mouse and human mLOY AML (Figure 2H and Supplemental Figure 6C). Notably, the genes significantly upregulated in the sg*Kdm5d* HSPCs were positively enriched in both AML patients and mice with mLOY, while those significantly downregulated in the sg*Kdm5d* HSPCs were negatively enriched in both mouse and human cells with mLOY (Supplemental Figure 6, D and E). Thus, *Kdm5d* loss partially resembled mLOY in HSPCs.

## Discussion

mLOY was first observed more than half a century ago (1). The following studies, especially recent genomics studies, provide accumulating evidence establishing mLOY as one of the most frequent chromosome alterations in humans (3, 30). More importantly, it is strongly associated with aging and numerous aging-related diseases, including cancer (4, 10, 12). In this study, we provide the first evidence to our knowledge that mLOY has a causal effect on AML and CH, the two most significant conditions associated with mLOY in elderly males. However, given the complexity of the cell populations involved in AML and CH, further studies are needed to dissect the effects of mLOY in different HSC, lineage-committed progenitors, and other populations in more details.

Given that many other human diseases, such as Alzheimer’s disease, cardiovascular events, and various solid cancers have also been associated with mLOY in patients and mLOY has also been detected in nonhematopoietic cells (37), it would be interesting to test whether mLOY also plays roles in other associated conditions.

Chromosome Y is a tiny chromosome with very few genes, and the synteny across species is low. Whether mLOY is a common feature among different species remains an open question. Our study suggests that mLOY in mice faithfully recapitulates the pathology and molecular features of human AML and CH with mLOY. Further, through bioinformatics and functional studies, we showed that *KDM5D* loss partially mediated mLOY in AML, CH, and DNA damage. KDM5D is a highly conserved and broadly expressed H3K4 demethylase (38). It would be interesting to identify the downstream targets of KDM5D in HSPCs and AML. KDM5D has been shown to prevent tumorigenesis, progression, and drug response in prostate cancer and gastric cancer (36, 39, 40). Whether it also plays a critical role in other mLOY-associated syndromes requires further study.

We find that mLOY cells have increased DNA damage, which phenocopies the genomic instability in human blood cells with mLOY (12). The mLOY- or *KDM5D* loss–induced genomic instability might explain the increased risk of tumorigenesis in elderly males with mLOY. Furthermore, defects in DNA damage response might predict a good prognosis for DNA damaging reagents (6, 15, 41). The distinct mechanisms of DNA damage responses in embryonic and somatic cells (22, 23) might explain the distinct phenotypes of XO mice previously reported (21) and mLOY mice generated in the current study. And more importantly, it provides a mechanism for the different disease spectrum of patients with Turner syndrome and those with mLOY (24, 25).

### Conclusions.

Taken together, these results indicate that mLOY in HSPCs promotes AML and CH, partially through loss of *Kdm5d*.

## Methods

### Mice.

*Trp53*^–/–^ mice (stock 002101), Rosa-Cas9 mice (stock 024858), and mCD45.1 mice (stock 002014) were from The Jackson Laboratory. By breeding Rosa-Cas9 mice with *Trp53*^–/–^ mice or mCD45.1 mice, we generated *Trp53*^–/–^; Cas9 mice or mCD45.1; Cas9 mice. Bone marrow cells were enriched from 8-week-old *Trp53*^–/–^; Cas9 male mice or mCD45.1; Cas9 male mice. c-Kit^+^ HSPCs were purified by autoMACS (Miltenyi Biotec) with mouse CD117 MicroBeads (Miltenyi Biotec, catalog 130-091-224). Purified HSPCs were cultured in BCM medium (50% DMEM + 50% IMDM) supplemented with 20% FBS, 2 ng/mL mouse IL-3 (R&D Systems, catalog 403-ML-050), 10 ng/mL mouse IL-6 (R&D Systems, catalog 406-ML-200), and 2 ng/mL mouse stem cell factor (SCF) (R&D Systems, catalog 455-MC-010). Retroviruses carrying *AML1-ETO* or truncated-*Kdm5d* cDNA and lentiviruses carrying sgRNA were introduced by calcium phosphate–mediated transfection of 293T packaging cells. HSPCs were transfected by spinoculation. For in vivo experiments, infected HSPCs (1 × 10^6^) were transplanted into sublethally irradiated (5.5 or 7 Gy) C57BL/6 female recipient mice by tail vein injection. For the AML cell transplant experiments, 1 × 10^6^ bone marrow cells were transplanted into sublethally irradiated C57BL/6 female recipients. Mice were monitored for clonal hematopoiesis or leukemia by CBC assay, blood smear staining, and flow cytometry. Mice were sacrificed and analyzed upon being moribund. Statistical analysis of all survival data was accomplished with the log-rank test in GraphPad Prism (RRID: SCR_002798).

### Plasmid constructs.

sgRNAs were cloned into the pLentiCRISPR-mCherry vector(U6-sgCas9-U6-sgRNA-EFS-mCherry). The target sequence of Cas9 is GATCGGCGACCAGTACGCC; the target sequences of *Ssty2* are *Ssty2*-A ATCACTCAAGAAGAAGAGT and *Ssty2*-B GGAGCTCCACAGCGATGAG; the target sequences of *Ssty1* are *Ssty1*-A ATCCCTCATGAAGAAGAGG and *Ssty1*-B GGAGCTCTACAGTGATGAC; and the target sequences of *Kdm5d* are *Kdm5d*-A ATGGTACCTACAGAAGTTG and *Kdm5d*-B GACTTATCTCCTGAAGAAA. The control scrambled CRISPR sequence is ACATTTCTTTCCCCACTGG, which allowed excision of the non-gene region on mouse chromosome 8.

The truncated *Kdm5d* (gene ID:20592) gene produces a 659–amino acid protein product (aa 1–695, including the JmjN, ARID, PHD-type 1, and JmjC domains). *Kdm5d* cDNA was cloned by PCR from the central nervous system cDNA library of an E14.5 male mouse embryo. The truncated *Kdm5d* cDNA was cloned into retroviral constructs (MSCV-truncated *Kdm5d*-IRES-GFP), as was *AML1-ETO* (MSCV-*AML1-ETO*-IRES-GFP).

### FISH.

Cells were incubated in 0.075 M KCl and then fixed in 3:1 methanol/glacial acetic acid (v/v) and dropped onto microscope slides. The slides were incubated at 55°C for 30 minutes, followed by the addition of 1 μL mouse chromosome Y control probe (Empire Genomics, catalog MCENY-10-GR) and 1 μL mouse chromosome XqA7.3 probe (Guangzhou Exon Biotechnology, catalog XY-105) to each slide. The probes were hybridized in a Hybridization Instrument (SH2000, Hangzhou Ruicheng Instrument Co.,Ltd) at 72°C for 5 minutes and then 37°C overnight. Slides were rinsed for 2 minutes in 0.3% NP-40/2× SSC at 72°C and 1 minute in 0.1% NP-40/2× SSC at room temperature. Finally, the slides were stained with 10 μL DAPI-antifade solution and mounted with a coverslip. The samples were captured using an Olympus BX53 fluorescence microscope.

### Comet assay.

HSPCs (0.5 × 10^4^) were mixed with 140 μL of 1% low-melting-point agarose, and 2 drops were placed on a precoated slide and incubated at 4°C for 5 minutes. Slides were then submerged in cold working alkaline lysis buffer overnight at 4°C, followed by alkaline unwinding for 40 minutes in cold alkaline electrophoresis buffer. The samples were resolved by electrophoresis at 25 V and 300 mA for 50 minutes at 4°C, after which the gels were neutralized in neutralization buffer for 30 minutes at 4°C. The sample images were captured using an Olympus BX53 fluorescence microscope after SYBR Green staining. The relative grayscale of the tail moment was analyzed by ImageJ (RRID: SCR_003070).

### Immunofluorescence.

Cytospins were prepared from HSPCs or tumor cell suspensions. The slides were air dried for 1–3 minutes and fixed with 4% paraformaldehyde solution for 30 minutes. The solution was removed, and the slides were washed 3 times with PBS, permeabilized with 0.3% Triton X-100 for 15 minutes, and then washed 3 times with PBS. Each slide was covered with 1% BSA and glutamic acid in PBST overnight at 4°C. Then the slides were incubated with antibodies against phospho-histone H2A.X (Ser139) (Cell Signaling Technology, catalog 9718S) or Cas9 (*S*. *pyogenes*) (E7M1H) XP rabbit mAb (Cell Signaling Technology, catalog 19526S) overnight at 4°C, dissolved 1:500 or 1:100 in 1% BSA in PBS. After washing the slides 3 times with PBS for 5 minutes each, they were incubated for 1 hour with goat anti-rabbit Alexa Fluor 633 diluted 1:1000 in PBS containing 1% BSA, followed by washing 3 times with PBS for 5 minutes each. Finally, the slides were stained with 10 μL DAPI-antifade solution and mounted with a coverslip. The sample images were captured using a ZEISS LSM880 confocal microscope. Statistical analysis of all data was accomplished with Imaris (RRID: SCR_007370).

### Flow cytometry.

Peripheral blood was obtained from retro-orbital puncture, and RBCs were lysed by ammonium chloride/potassium bicarbonate buffer. Antibody staining was performed at 4°C for 30 minutes with PBS supplemented with 2% FBS. Flow cytometry was performed with the following antibodies (all from BioLegend): PE/Cyanine 7 anti–mouse CD117 (c-Kit) (catalog 105814), Pacific Blue anti–mouse/human CD11b (Mac1) (catalog 101224), Pacific Blue anti–mouse Ly-6G/Ly-6C (Gr-1) (catalog 108430), APC anti–mouse/human CD45R/B220 (B220) (catalog 103212), and APC anti–mouse CD3ε (CD3) (catalog 100312). Flow cytometric analysis was performed using FlowJo (RRID: SCR_008520), and flow cytometry was performed on an LSRFortessa (BD Biosciences).

### RNA-seq analysis.

RNA was extracted from AML tumor cells (bone marrow cells or splenocytes) or HSPCs with the RNeasy Mini Kit (Qiagen, catalog 74104) following the manufacturer’s instructions. Each group consisted of 3 replicates. RNA quality was analyzed using Agilent picochips. Samples with an RNA integrity number (RIN) of 7.5 or greater were analyzed by RNA-seq. RNA libraries were prepared according to the standard Illumina protocols and used for sequencing. The RNA-seq data were sequenced using an Illumina NovaSeq 6000, and 150-bp paired-end reads were obtained. The company carried out preliminary quality control of the raw data, removing the adapter, poly-N, and low-quality reads to obtain clean data.

The mouse RNA-seq reads were aligned to the reference genome GRCm38 by STAR (42). Transcripts were normalized by DESeq2 (43). Genes with 2-fold up- or downregulation and FDR of 0.05 or less were identified as differentially expressed genes. GSEA used statistical approaches to identify significant similarities and differences between 2 given clusters by identifying a priori–defined gene sets (44). Differentially expressed genes of the sg*Kdm5d* group were compared with the sgScr group using heatmaps constructed by pheatmap (https://www.rdocumentation.org/packages/pheatmap/versions/0.2/topics/pheatmap) and standardized gene expression determined by DESeq2 (43) was normalized by *z* score. The intersection of downregulated genes in mLOY patients and mLOY mice was analyzed by VennDiagram (45).

RNA-seq data of AML patients were downloaded from TARGET AML, TCGA LAML, and BEAT AML (32), transcripts were normalized by DESeq2 (43), and differential expression analysis was performed. The expression of *KDM5D* compared with WT in mLOY patients and mLOY mice was visualized by ggpubr (https://rpkgs.datanovia.com/ggpubr/). We used ggplot2 to construct the pie chart of the proportion of mLOY patients among AML patients and mLOY patients among AML patients with *AML1-ETO*.

### Data availability.

All RNA-seq data used in this study have been deposited in NCBI’s Gene Expression Omnibus database (GEO GSE165208).

### Statistics.

All data are presented as the mean ± SD, unless otherwise indicated. For comparisons of 2 groups, the 2-tailed, unpaired *t* test and 2-tailed Mann-Whitney test were performed. For comparisons of 3 or more groups, 1-way ANOVA was performed with the Benjamini-Hochberg multiple-comparison test, 2-way ANOVA was performed with Sidak’s multiple-comparison test, and Kruskal-Wallis test was performed with the Benjamini-Hochberg multiple-comparison test. For Kaplan-Meier tumor-free survival curves, log-rank tests were performed. All statistics are indicated for each figure and analyses were conducted using GraphPad Prism 8. NS, not significant; *P < 0.05 or FDR *q* < 0.05; **P < 0.01 or FDR *q* < 0.01; ***P < 0.001 or FDR *q* < 0.001, ****P < 0.0001 or FDR *q* < 0.0001, as indicated in the figure legends.

### Study approval.

All mouse experiments were approved by the Institutional Animal Care and Use Committee of of West China Hospital of Sichuan University (approval number 2021822A).

## Author contributions

YL and CC conceived the project and wrote the manuscript. QZ performed experiments, analyzed data, and wrote the manuscript. LZ performed bioinformatics analysis and wrote the manuscript. YY and SL performed DNA damage assays.

## Supplementary Material

Supplemental data

## Version 1 02/08/2022

Electronic publication
