# Supplementary material for: Mosaic loss of chromosome Y promotes leukemogenesis and clonal hematopoiesis
Source: JCI Insight. 2022 Feb 8;7(3):e153768. doi: 10.1172/jci.insight.153768 (PMC8855789; doi:10.1172/jci.insight.153768)
Supplement: Supplemental data [file jciinsight-7-153768-s005.pdf]

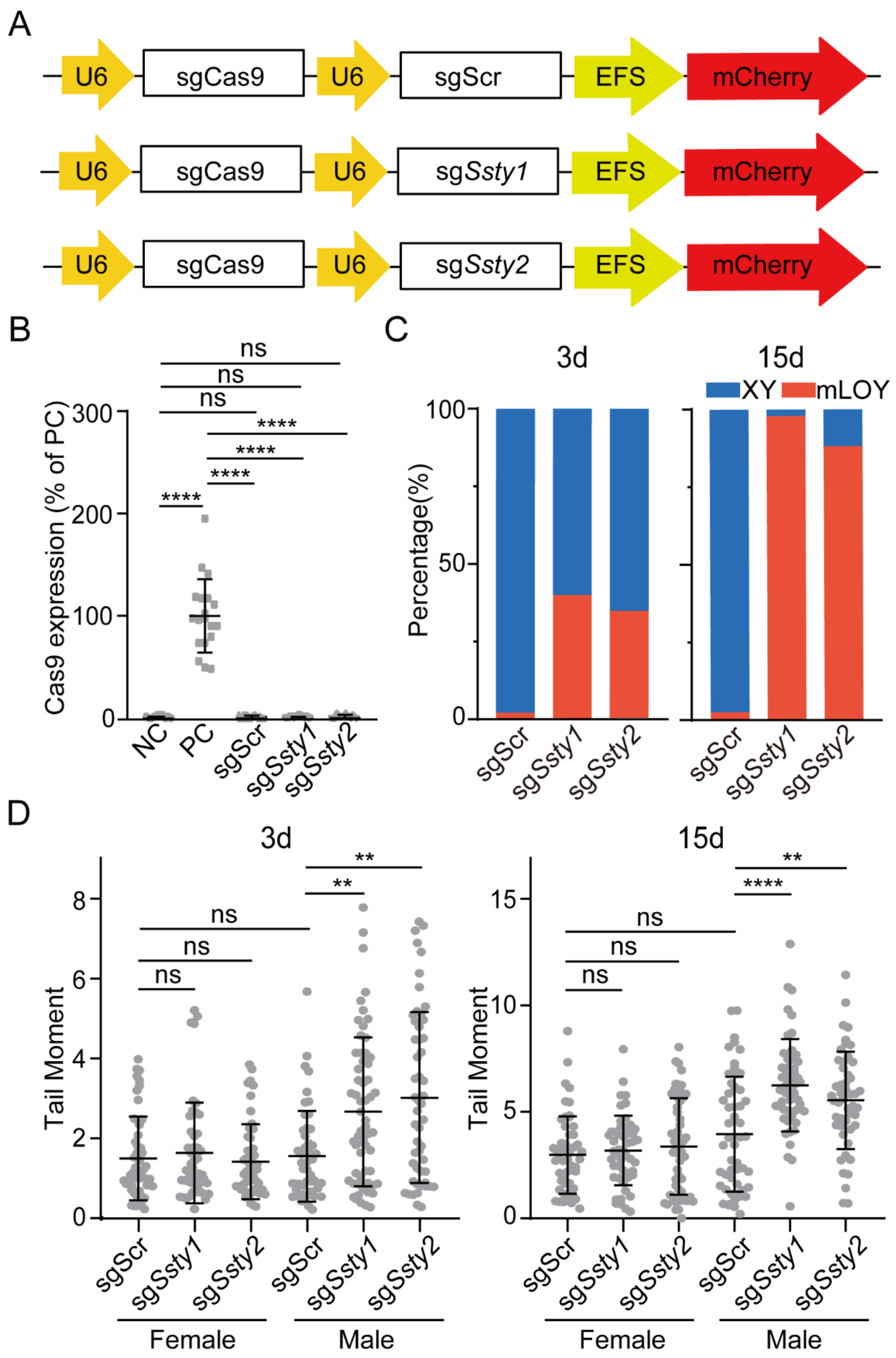

Supplementary Figure 1. A suicide sgCas9 depleted Cas9 in HSPCs.

A. Schematic representation of lentivirus vector expressing mCherry, U6-sgRNA

targeting Y chromosome (sg*Ssty1*, sg*Ssty2*) connected with U6-sgCas9 in one vector, U6-sgCas9-U6-sgScr was used as negative control. **B**. Statistical graph of Cas9 expression by immunofluorescent in HSPCs from WT mice (NC), HSPCs from Cas9 mice (PC), HSPCs from Cas9 mice with sgScr-sgCas9 (sgScr), sg*Ssty1*-sgCas9 (sg*Ssty1*) or sg*Ssty2*-sgCas9 (sg*Ssty2*). Statistical analysis represented on graph is relative to positive control (PC). ns, not significant; **** FDR q<0.0001 (Kruskal-Wallis test). **C**. Statistical graph of the percentages of mLOY in mCherry+ sgScr-sgCas9, sg*Ssty1*-sgCas9 and sg*Ssty2*-sgCas9 HSPCs at 3 days (left) and 15 days (right) after infection. **D**. HSPCs from female and male mice were infected with sgScr-sgCas9, sg*Ssty1*-sgCas9 or sg*Ssty2*-sgCas9. Comet assay of mCherry+ HSPCs was check at 3 days (left) and 15 days (right) after infection. The tail moment was shown as the means ± SD. ns, not significant, ** FDR q <0.01, **** FDR q <0.0001 (Kruskal-Wallis test).

A

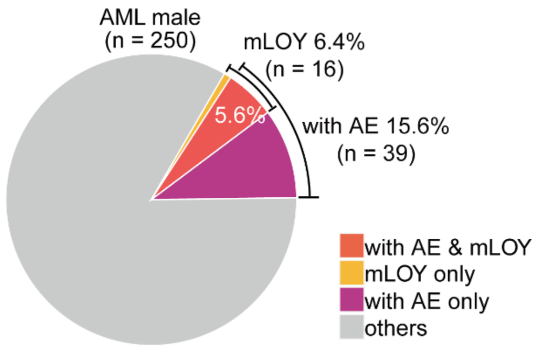

	mLOY -	mLOY +	All
AE -	209	2	211
AE +	25	14	39
All	234	16	250

p-value: 2.6e - 16

B

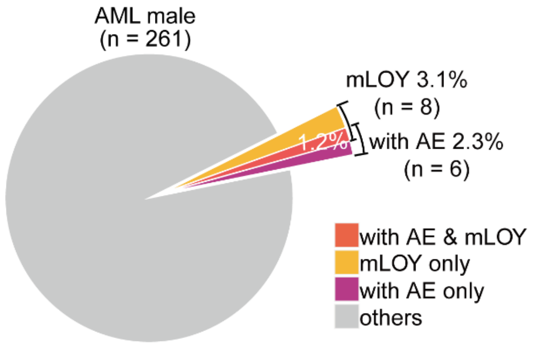

	mLOY -	mLOY +	All
AE -	250	5	255
AE +	3	3	6
All	253	8	261

p-value: 1.5e - 11

C

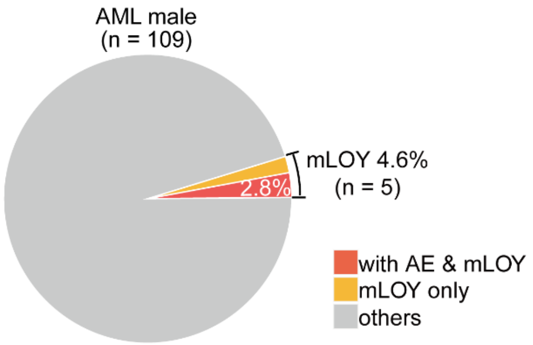

	mLOY -	mLOY +	All
AE -	104	2	106
AE +	0	3	3
All	104	5	109

p-value: 1.1e - 15

Supplementary Figure 2. mLOY was associated with *AML1-ETO* in AML.

A-C. Co-occurrence of *AML1-ETO* with mLOY in male AML patients. Pie charts showing ratios of mLOY and *AML1-ETO* in all-male AML patients from the TARGET AML cohort (A, $p < 2.6e-16$, Chi-square test), BEAT AML cohort (B, $p < 1.5e-11$, Chi-square test) and TCGA LAML cohort (C, $p < 1.1e-15$, Chi-square test).

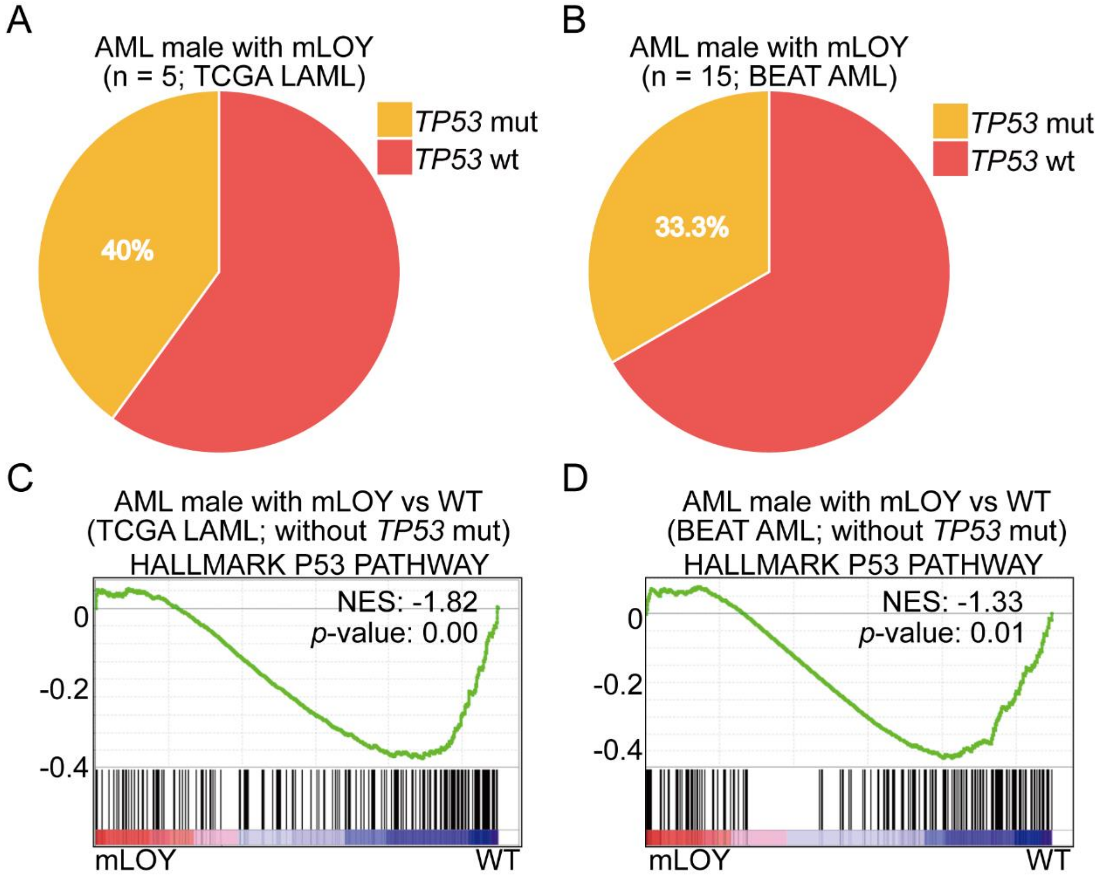

Supplementary Figure 3. **mLOY was associated with *TP53* mutations and/or downregulation of the p53 pathway.**

A-B. Pie charts showing ratios of *TP53* mutation in all mLOY AML male patients (A, TCGA LAML cohort; B, BEAT AML cohort). **C-D.** GSEA showing the negative enrichment of the HALLMARK_P53_PATHWAY in p53 intact AML patients with mLOY (C, TCGA LAML cohort; D, BEAT AML cohort).

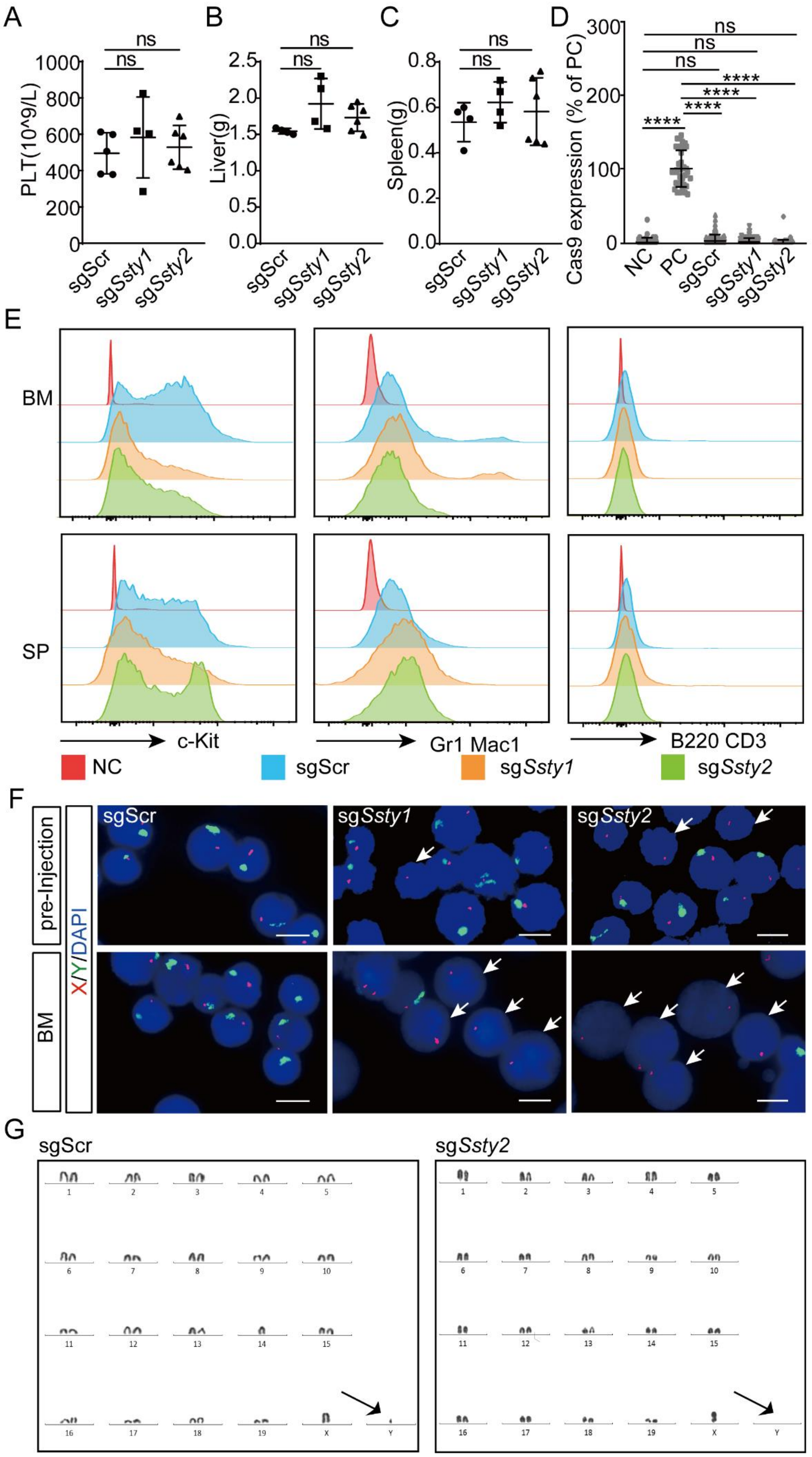

A
B
C
D
ns
ns
PLT(10^9/L)
Liver(g)
Spleen(g)
Cas9 expression (% of PC)
****
sgScr
sgSsty1
sgSsty2
NC
PC
E
BM
SP
c-Kit
Gr1 Mac1
B220 CD3
NC
sgScr
sgSsty1
sgSsty2
F
pre-Injection
BM
X/Y/DAPI
G

Supplementary Figure 4. The role of mLOY in *AML1-ETO+* AML.

A. Number of platelet (PLT) in recipient mice at 7 weeks after transplantation, measured by complete blood cell counts. n=5,4,6. ns, not significant (One-way ANOVA). **B-C.** Weight of liver(B, n=4,4,6) and spleen(C, n=4,4,6) in transplanted AML mice. Data are showing means SD. ns, not significant (One-way ANOVA). **D.** Statistical graph of Cas9 expression by immunofluorescent of cells isolated from WT mice spleen (NC), Cas9 mice spleen (PC), sgScr tumor cells(sgScr) and mLOY tumor cells (sg*Ssty1* and sg*Ssty2*) with sgCas9. The statistical analysis represented on the graph is relative to a positive control (PC). 3 recipient mice were measured for each group. ns, not significant; **** FDR q <0.0001 (Kruskal-Wallis test). **E**. Flow cytometry analyses of the surface marker (c-kit; Gr1 and Mac1;B220 and CD3) of tumor cells from bone marrow (top) and spleen (bottom) in sgScr-sgCas9, sg*Ssty1*-sgCas9 and sg*Ssty2*-sgCas9 AML mice. **F.** Representative photomicrograph of FISH in sgScr-sgCas9;*AML1-ETO*, sg*Ssty1*-sgCas9;*AML1-ETO* and sg*Ssty2*-sgCas9;*AML1-ETO* HSPCs before injection (top) and blast cells in AML mice BM (bottom). Green, FITC-labeled whole-chromosome probe for Y chromosome; red, Texas red-labeled X chromosome probe for XqA7.3; blue, DAPI-labeled DNA. White arrows indicate XO cells, bar, 10μm. **G.** Cytogenetic analysis of splenocytes from sg*Ssty2*-sgCas9; *AML1-ETO* AML mice. Black arrows indicate chromosome Y.

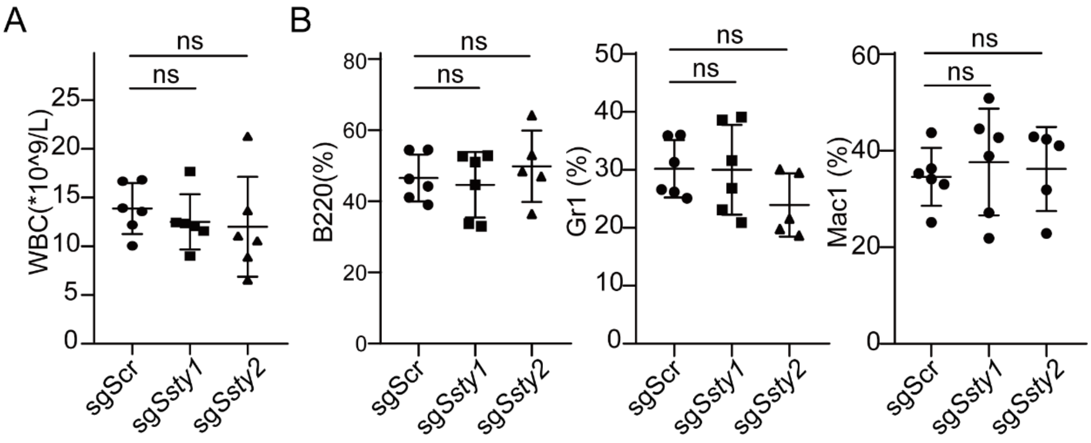

Supplementary Figure 5. **The effect of mLOY on hematopoiesis in mice.**

A. White blood cell counts (WBC) of recipient mice at 9 weeks after transplant with sgScr-sgCas9, sg*Ssty1*-sgCas9, sg*Ssty2*-sgCas9 HSPCs, showing as means ± SD. n=6, ns, not significant (One-way ANOVA). **B.** Percentages of B220+(left, n=6,6,5), Gr1+(middle, n=6,6,5), and Mac1+(right, n=6,6,5) cells in PB of recipient mice transplanted with sgScr-sgCas9, sg*Ssty1*-sgCas9 and sg*Ssty2*-sgCas9 HSPCs, measured by flow cytometry, ns, not significant (One-way ANOVA).

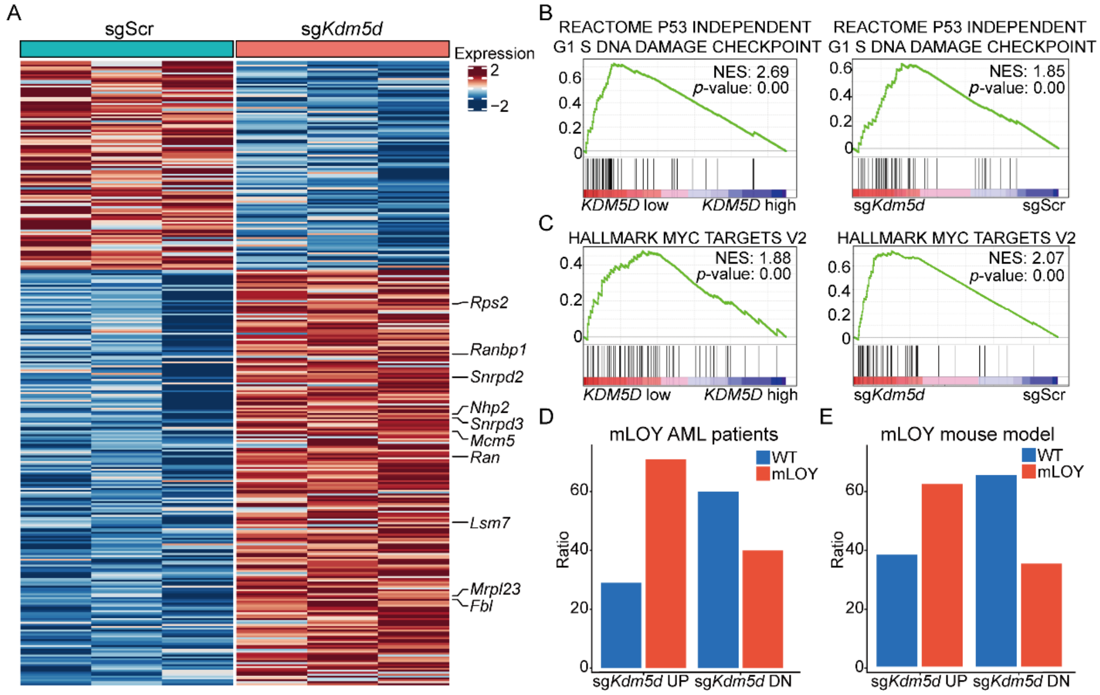

Supplementary Figure 6. The transcriptomic analyses of *KDM5D* deficient cells.

A. Heatmap showing the differentially expressed genes (DEGs) in sg*Kdm5d* HSPCs compared to those with sgScr. **B.** GSEA showing the positive enrichment of the Reactome_P53_independent_G1_S_DNA_damage_checkpoint pathway in AML patients with *KDM5D* low expression and sg*Kdm5d* HSPCs. **C.** GSEA showing the positive enrichment of Hallmark_MYC_targets_V2 pathway in AML patients with *KDM5D* low expression and sg*Kdm5d* HSPCs. **D-E**. The proportion of sg*Kdm5d* HSPC UP and DOWN signatures (top200 significantly up-and down-regulated genes) in the mLOY AML patients (D) and mLOY AML mouse model (E) compared with control.

Cytogenetics

	AML1-ETO	PML-RARA	Complex Cyto.	Normal Karyotype	other fusion	others	All
KDM5D low	0	3	7	14	6	12	42
KDM5D high	3	4	4	16	9	5	41
All	3	7	11	30	15	17	83

Supplementary Table 1. The cytogenetics of *KDM5D* low and *KDM5D* high patients in the TCGA LAML cohort.
